# Analysis of whole Y-chromosome sequences reveals the Japanese population history in the Jomon period

**DOI:** 10.1038/s41598-019-44473-z

**Published:** 2019-06-17

**Authors:** Yusuke Watanabe, Izumi Naka, Seik-Soon Khor, Hiromi Sawai, Yuki Hitomi, Katsushi Tokunaga, Jun Ohashi

**Affiliations:** 1 Department of Biological Sciences, Graduate School of Science, The University of Tokyo, Tokyo, 113-0033 Japan; 2 Department of Human Genetics, Graduate School of Medicine, The University of Tokyo, Tokyo, 113-0033 Japan

**Keywords:** Anthropology, Population genetics

## Abstract

The Jomon and the Yayoi are considered to be the two major ancestral populations of the modern mainland Japanese. The Jomon people, who inhabited mainland Japan, admixed with Yayoi immigrants from the Asian continent. To investigate the population history in the Jomon period (14,500–2,300 years before present [YBP]), we analyzed whole Y-chromosome sequences of 345 Japanese males living in mainland Japan. A phylogenetic analysis of East Asian Y chromosomes identified a major clade (35.4% of mainland Japanese) consisting of only Japanese Y chromosomes, which seem to have originated from indigenous Jomon people. A Monte Carlo simulation indicated that ~70% of Jomon males had Y chromosomes in this clade. The Bayesian skyline plots of 122 Japanese Y chromosomes in the clade detected a marked decrease followed by a subsequent increase in the male population size from around the end of the Jomon period to the beginning of the Yayoi period (2,300 YBP). The colder climate in the Late to Final Jomon period may have resulted in critical shortages of food for the Jomon people, who were hunter-gatherers, and the rice farming introduced by Yayoi immigrants may have helped the population size of the Jomon people to recover.

## Introduction

Archaeological studies have shown that the Japanese prehistory is divided into three periods: the Paleolithic period (older than 14,500 years before present [YBP]), the Jomon period (14,500-2,300 YBP), and the Yayoi period (2,300-1,700 YBP)^1^. One of the most important events in Japanese history is the transition from subsistence food production activities such as hunting and gathering to farming rice at the beginning of the Yayoi period. The advanced rice farming is thought to have been introduced to mainland Japan (we use the term “mainland Japan” to distinguish Honshu, Shikoku and Kyusyu from Hokkaido and Okinawa in this paper), inhabited by the Jomon people, by the Yayoi immigrants who came from the Asian continent.

**Figure 1:**
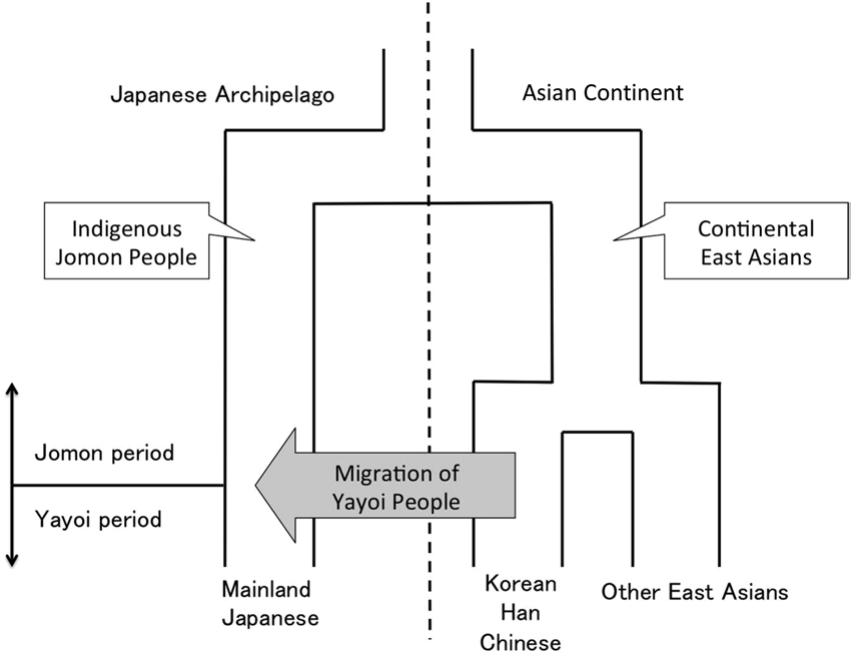
Overview of the currently accepted demographic model of the mainland Japanese population.

**Figure 2:**
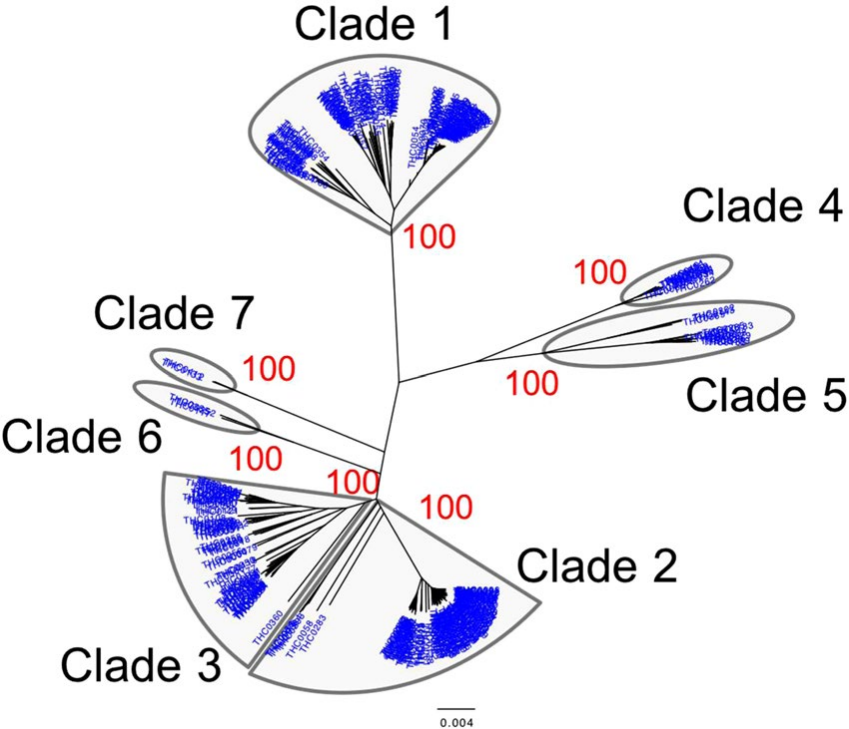
Neighbor-joining tree of mainland Japanese Y chromosomes. The neighbor-joining tree of 345 mainland Japanese Y chromosomes was constructed using MEGA7 based on 28,254 SNPs.

At present there are two minor populations and one major population in the Japanese archipelago: the Ainu who mainly live in Hokkaido (a minor population); the mainland Japanese (the major population); and the Ryukyuan who mainly live in Okinawa (a minor population). The prevailing hypothesis on the origins of the Japanese is “the dual structure model,^2^” where the present-day Japanese population is formed by an admixture between indigenous Jomon people and Yayoi immigrants who migrated from continental East Asia (Fig. 1). According to this model, modern mainland Japanese should have genomic components derived from both the Jomon people and those originating from the Yayoi immigrants. Genetic studies, using genome-wide single nucleotide polymorphism (SNP) data, basically supported the above model^3–6^. A recent study of ancient DNA extracted from the remains of Jomon individuals who had lived in mainland Japan (Fukushima Prefecture) 3,000 YBP suggested that (1) the Jomon people were strongly divergent from the current East Asians (Fig. 1), (2) the Ainu people are most closely related to the Jomon people among the three Japanese populations, and (3) ~12% of the genomic components of the modern mainland Japanese were derived from the Jomon people^7^. Genetically, the mainland Japanese are closely related to Koreans, followed by Han Chinese, and finally by other Continental East Asians^3^. These findings suggest that a large number of Yayoi immigrants came mainly through the Korean Peninsula to mainland Japan, and the mainland Japanese still retain genomic components from the Jomon people. Thus, there is a possibility that the historical change in the population size of the historic Jomon people, who lived in mainland Japan, could be estimated from genomic data from the modern mainland Japanese.

In the current work, we estimated the change in population size of the Jomon people in the Late to Final Jomon period by using whole Y-chromosome sequences of Japanese males living in mainland Japan. Specifically, we identified a phylogenetic clade (hereinafter called “clade 1”) specific to Japanese Y chromosomes and largely divergent from ones attributable to the continental East Asians. Y chromosomes in clade 1 appeared to be derived from the Jomon people. The population frequency of clade 1 Y chromosomes was estimated to be ~70% in Jomon males. The Bayesian skyline plot (BSP)^8^ for Y chromosomes in clade 1 allowed us to infer the historical change in population size of the majority of the Jomon people. Our results suggested that the Jomon people underwent a major decrease in population size at the end of the Jomon period, with a recovery occurring soon after the beginning of the Yayoi period.

## Results

### Phylogenetic Tree of Y Chromosomes

**Figure 3:**
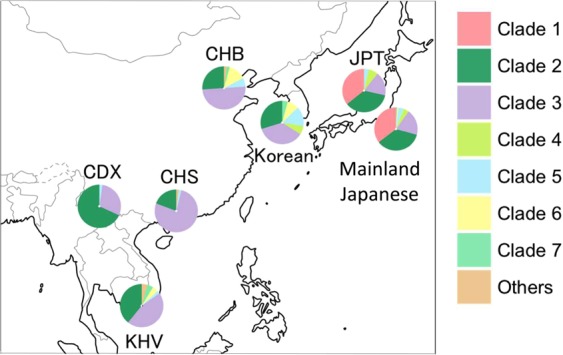
Frequencies of seven Y-chromosomal clades in East Asian populations. Y chromosomes in clade 1 were observed only in the Japanese among the East Asian populations. Frequencies of five East Asian populations were obtained from the 1000 Genomes Project phase 3 data. JPT: Japanese in Tokyo, Japan; CHB: Han Chinese in Beijing, China; CHS: Southern Han Chinese; CDX: Chinese Dai in Xishuangbanna, China; and KHV: Kinh in Ho Chi Minh City, Vietnam.

A total of 14 Mb of reliable sites had a call rate greater than 95% on the whole Y-chromosome sequences of 345 Japanese males (depth: 21×), and 28,254 Y-chromosomal SNPs were identified. Initially, a phylogenetic analysis was conducted for 345 Y chromosomes using the 28,254 SNPs. The Japanese Y chromosomes were divided into seven distinct clades in the neighbor-joining (NJ)^9^ tree (Fig. 2). To further investigate the phylogenetic relationship of East Asian Y chromosomes, a NJ tree of Y chromosomes from 345 Japanese, 47 Koreans^10^, and 244 East Asians^11^ was constructed using the 3,006 SNPs (Fig. S1). Although the branch lengths of the NJ tree in Fig. S1 were not correct because of the removal of population-specific variants, the topology was correct. We found a clade that contains only Japanese Y chromosomes in Fig. S1. Table 1 and Fig. 3 show the population frequency of each clade in East Asians. In this study, Japanese analyzed in 1000 Genomes Project are labeled “JPT” (“Japanese in Tokyo”) to distinguish our subjects from mainland Japanese. Incidentally, a total of 838 mitochondrial SNPs were identified from the same subjects (345 Japanese males). However, in contrast to the situation with the Y chromosomes, no clade specific to the Japanese population was found in the NJ tree of mtDNA sequences (Fig. S2).

**Table 1:** Frequencies of seven Y chromosomal clades in East Asian Populations.

	Clade 1	Clade 2	Clade 3	Clade 4	Clade 5	Clade 6	Clade 7
Mainland Japanese	0.354	0.354	0.197	0.041	0.041	0.009	0.006
Korean	0	0.298	0.362	0.064	0.149	0.085	0.043
JPT	0.357	0.357	0.179	0.071	0.036	0	0
CHB	0	0.261	0.500	0	0.065	0.130	0.022
CHS	0	0.192	0.769	0	0.019	0	0
CDX	0	0.682	0.295	0	0.023	0	0
KHV	0	0.391	0.457	0	0.022	0.043	0.043

Frequency of five East Asian populations were obtained from the 1000 Genomes project phase 3 data. JPT: Japanese in Tokyo, Japan, CHB: Han Chinese in Beijing, China, CHS: Southern Han Chinese, CDX: Chinese Dai in Xishuangbanna, China, and KHV: Kinh in Ho Chi Minh City, Vietnam.

### Y-Chromosome Clades in the Mainland Japanese

The population frequency of Y chromosomes in clade 1 was 35.4% in the mainland Japanese and 35.7% in JPT (Table 1). The genotyping of rs2032626 (rs2032626-G allele is a marker for haplogroup D1b containing YAP+ variant) confirmed that clade 1 corresponded to haplogroup D1b (Fig. S3). Since the population frequencies of Y chromosomes in clades 2 and 3 were observed at relatively high frequencies in East Asians including Japanese (Table 1), a proportion of them seem to have been introduced to the mainland Japanese by Yayoi immigrants. The analysis of SNP markers revealed that Y chromosomes in clades 2 and 3 corresponded to haplogroups O1 and O2, respectively. Haplogroups O1 and O2 have been reported to be frequently observed in East Asians and Southeast Asians^12^. Clades 4 and 5 were assigned to haplogroup C, and clades 6 and 7 were assigned to haplogroup N.

### Frequencies of Seven Y-Chromosome Clades in the Jomon People

Y chromosomes in clade 1 were absent from continental East Asians except for Japanese (Table 1), and the frequency of haplogroup D1b (i.e. clade 1) was reported to be very high (81.3%) in the Ainu^12^, who have been shown, of the three Japanese populations, to be genetically and morphologically most closely related to the Jomon people^7,13^. These results strongly suggest that the mainland Japanese Y chromosomes in clade 1 originated from the Jomon people. The frequency of clade 1 in the mainland Japanese population (35.4% in the mainland Japanese and 35.7% in JPT) was much larger than the proportion (12%) of the genomic components of the Jomon people in the modern mainland Japanese, estimated previously by a study of ancient DNA from Jomon individuals^7^. Thus, the population frequency of clade 1 Y chromosomes must have been much higher in the Jomon people than in the present mainland Japanese.

**Figure 4:**
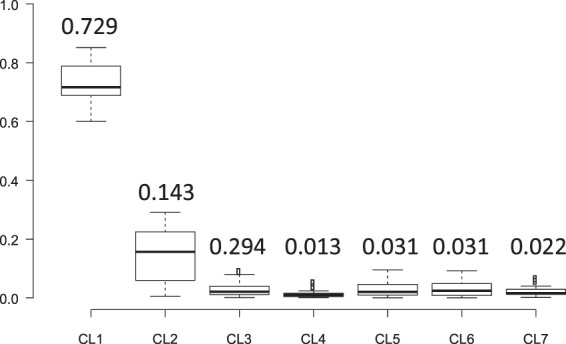
Estimated frequency of each clade in the Jomon people by Monte-Carlo simulation. Twenty sets of clade frequencies were selected from 10^8^ simulated data sets in ascending order of Similarity Index (*SI*). The mean frequency of 20 sets in each clade is shown above the box.

It was not possible to analytically estimate the population frequencies of seven clades in Jomon people. Instead, to confirm that the majority of Jomon people had clade 1 Y chromosomes, Monte-Carlo simulation, in which the population frequencies of the seven clades in the Jomon people are given by random numbers, was performed (see Material and Methods for details). The clade frequencies in an admixed population were calculated from those in the Jomon population (simulated) and those in Yayoi immigrants (assumed to be Koreans), and then compared with the clade frequencies observed in the current mainland Japanese (our subjects). The distribution of similarity index (*SI*) for 10^8^ sets of the clade frequencies is shown in Fig. S4. In this analysis, *SI* decreased, as the difference between the simulated and observed clade frequencies in the mainland Japanese diminished. Only 20 sets (*SI* <3.24) were selected from 10^8^ sets in order of increasing *SI* (Fig. 4), and the mean values of the population frequencies of Y chromosomes in clades 1, 2, and 3 were 73%, 14%, and 2%, respectively. This result implies that ~70% of the Jomon people in mainland Japan possessed clade 1 Y chromosomes, whereas the current mainland Japanese clade 3 Y chromosomes were mostly derived from Yayoi immigrants.

### Bayesian Coalescent Inference of Historical Change in Population Size

**Figure 5:**
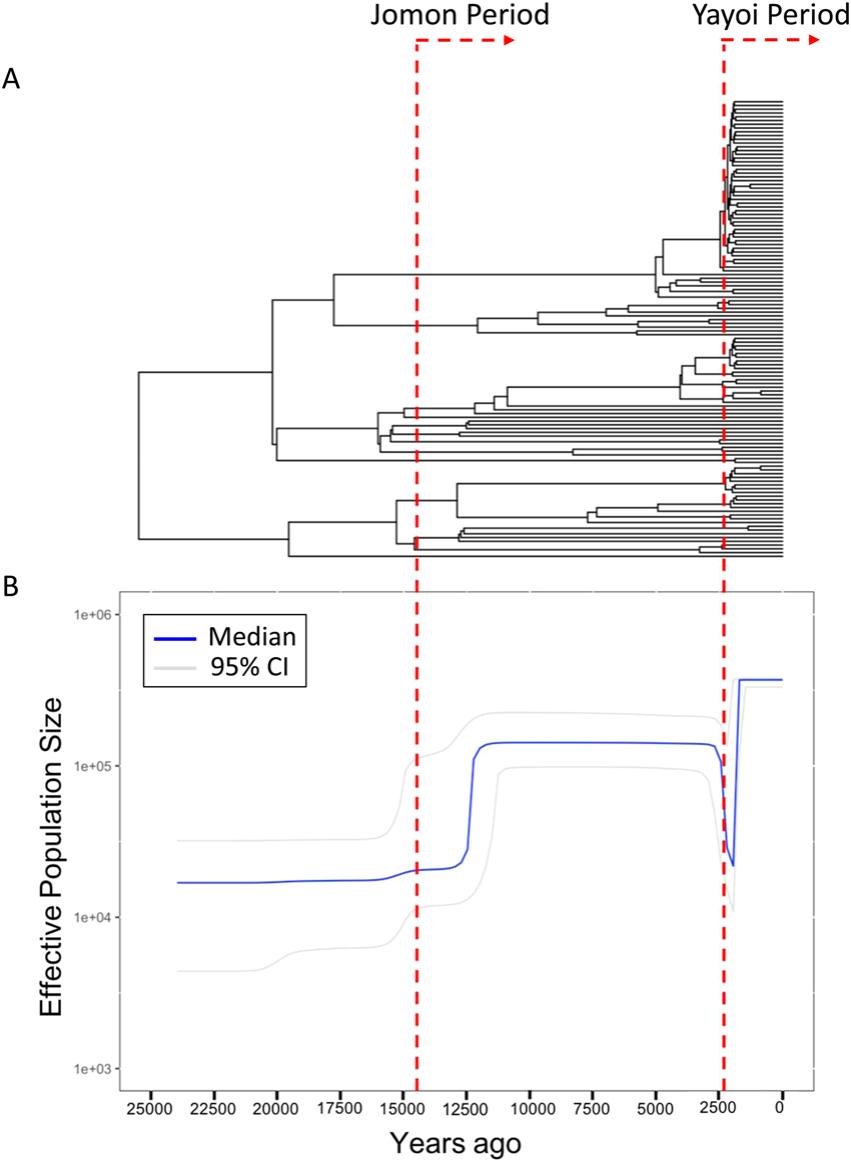
Phylogenetic tree and Bayesian skyline plot (BSP) of Y chromosomes of mainland Japanese in clade 1. (**A**) Phylogenetic tree of clade 1 estimated by BEAST 2.4.4. (**B**) BSP of 122 Y chromosomes of mainland Japanese in clade 1. BEAST 2.4.4 was used to obtain the plot. The red dashed lines represent 14,500 YBP (years before present; the beginning of the Jomon period) and 2,300 YBP (the beginning of the Yayoi period).

To deduce the historical change in the effective male population size of the Jomon people, Bayesian Skyline Plot (BSP) analyses^8^ were performed using BEAST 2.4.4 for 122 Y chromosomes of mainland Japanese belonging to clade 1 (Fig. 5). In the plot, the effective male population size started to increase gradually after the beginning of the Jomon period (12,500 YBP). Then, the population size decreased at 3,200 YBP (i.e. coalescent events frequently occurred 3,200 YBP as shown in Fig. 5A). Soon after the decrease, the effective population size of clade 1 rapidly increased to the present level. In addition, 68 Y chromosomes of the mainland Japanese in clade 3 were inspected to obtain information on the demography of a proportion of the Yayoi immigrants (Fig. S5). In contrast to the situation with clade 1, no population decrease was observed in the BSP of clade 3, implying that no severe bottleneck occurred when Yayoi immigrants came to the Japanese archipelago. This coincides with the previous observation that ~88% of the genomic components of the modern mainland Japanese were derived from the Yayoi immigrants^7^.

### Coalescent Simulation

**Figure 6:**
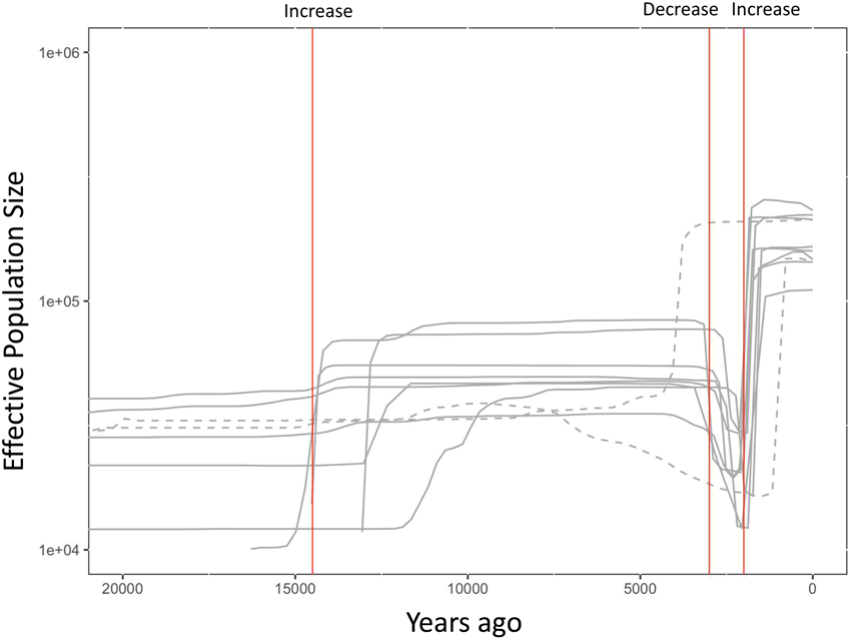
Bayesian skyline plots of major clades of simulated genealogies by Fastsimcoal2. BEAST 2.4.4 was used to obtain the BSPs for 200 simulated chromosomes only in a major clade produced by fastsimcoal2. The solid lines represent BSPs that detected the recent changes in population size (decrease of 3,000 YBP and increase of 2,000 YBP). The dashed lines represent BSPs that could not detect the population change assumed.

In this study, only clade 1 Y chromosomes were used to infer the historical change in population size of Jomon people. Although ~70% of Jomon males are considered to have possessed clade 1 Y chromosomes, the use of such biased samples may prevent us from accurately estimating the time of an historical event. To confirm that the BSP result of the major clade (clade 1) reflects the time when the change in population size occurred, we performed coalescent simulations by fastsimcoal2^14^, assuming that population size was changed three times (i.e., increase of 14,500 YBP, decrease of 3,000 YBP, and increase of 2,000 YBP). Then, the BSP was conducted for simulated chromosomes only in a major clade (frequency of more than 50%). The recent decrease and increase in the population size were successfully detected at the setting times (3,000 YBP and 2,000 YPB) in eight out of 10 simulation runs (Fig. 6, solid lines). As a consequence, the date at which the recent change in population size of Jomon population could be estimated by the BSP for Y chromosomes only in the major clade. However, considering the large difference in date of the population increase between the simulation results and the setting (14,500 YBP) in Fig. 6, the date of population increase around 12,500 YBP in Fig. 5 may not be reliable.

## Discussion

The present mainland Japanese have two ancestral populations: the indigenous Jomon and the Yayoi immigrants. In order to examine the demographic history during the Jomon period (14,500-2,300 YBP), we conducted a population genetic analysis using Y chromosomes of present-day mainland Japanese. In this study, a monophyletic group (named clade 1), consisting of only Japanese Y chromosomes, was identified in the phylogenetic analysis. Clade 1 was found to correspond to the Y– chromosome haplogroup D1b. This haplogroup, characterized by the insertion of an *Alu* element called YAP, is frequently observed in Japanese and Tibetans but is absent from other East Asians^12,15^. Although the lineages of haplogroup D1b in Japanese and Tibetans must have a common ancestor, the absence from Korean and Chinese populations suggests that the divergence of haplogroup D1b occurred before the ancestors of the Jomon people reached the Japanese archipelago. Y chromosomes in clade 1 are considered to have originated almost exclusively from the Jomon people who had inhabited the Japanese archipelago a long time before the Yayoi immigrants arrived. A simple computer simulation suggested that ~70% of the Jomon people carried the Y chromosome belonging to clade 1.

In BSP for Y chromosomes in clade 1, we observed significant decreases in the effective population size of the Jomon people at the end of the Jomon period (Fig. 5). In the Final Jomon Period, there was a cooling trend in Japan with a sea level 1–3 m lower than the present level^1^. For the first time, we provide genetic evidence that the number of Jomon people decreased simultaneously with the cooling in the Final Jomon Period. Since the Jomon people were hunter–gatherers dependent on wild food, the number of the Jomon people might have decreased due to the shortage of food resources in the colder Final Jomon Period. A previous study^16^ had reported that a dietary change occurred along with climate change during the Late to Final Jomon period. It has been revealed that the density of archaeological sites declined in the Late to Final Jomon period^17^, suggesting that a sizeable population decline had occurred in Japan. Our results strongly support the archaeological finding. The effective population size of the Jomon people was estimated to have increased after the beginning of the Yayoi period (i.e., 2,300 YBP) (Fig. 5). The livelihood of the mainland Japanese shifted from nomadic hunter-gatherer to settled agriculturist in the Yayoi period. The rice farming introduced to mainland Japan by Yayoi immigrants, therefore, may have helped the Jomon people to procure food stably and recover their population size.

Since it is known that effective male and female population sizes are different in general^18^ and the contributions of men and women are often unequal when admixture of two populations occurs^19^, the population history of Jomon females may be different from that of Jomon males. The ancient mtDNA analysis for Jomon people will add much to our understanding of historical changes in the effective female population size during the Jomon period.

## Materials and Methods

### Subjects

All 345 individuals investigated in this study were unrelated Japanese males living in Tokyo or neighboring areas. The genomic DNA was extracted from each peripheral blood sample using a commercial kit, QIAamp Blood Kit (Qiagen, Hilden, Germany). All blood and DNA samples were de-identified. Verbal informed consent was obtained from all the participants before 1990. In this study, written informed consent was not obtained because the blood sampling was conducted before the “Ethical Guidelines for Human Genome and Genetic Sequencing Research” were established in Japan. Under the condition that DNA sample is permanently de-linked from the individual, this study, including all experiments described in the Materials and Methods section was approved by the Research Ethics Committees of the Faculty of Science and of the Faculty of Medicine, University of Tokyo.

### Whole-Genome Sequencing

DNA samples were quantified with a Qubit 3.0 fluorometer using the Qubit dsDNA BR Assay Kit according to the manufacturer’s protocol. Degradation of DNA was examined using the Agilent 4200 TapeStation system (Agilent Technologies) according to the manufacturer’s protocol. The genomic DNA samples were diluted to a concentration of 20 ng/μl (1,100 ng) and fragmented in a 96-well plate using a DNA sonication system (Covaris LE220; Covaris) to an appropriate size for the following process. The sheared DNA was subjected to library construction with the TruSeq DNA PCR-Free LT sample prep kit (Illumina) following the manufacturer’s protocol for 350-bp insert size. The library quality control was performed with Agilent 4200 TapeStation (Agilent Technologies) using the High-Sensitivity D5000 Kit, according to the manufacturer’s protocol. The library quantification was performed with quantitative PCR using KAPA library quantification kit (KAPA Biosystems), according to the manufacturer’s protocol.

DNA libraries were analyzed using the HiSeq X sequencing system (Illumina), according to the manufacturer’s protocol. The libraries were diluted to the appropriate concentrations for sequencing and were used for cluster generation on an Illumina cBot cluster generation system with HiSeq X PE Cluster Kits v2 (Illumina). Individual libraries were clustered on a single lane of a HiSeq X Patterned flow cell. The clustered flow cell was sequenced on the Illumina HiSeq X Sequencing System with settings for generation of 2 × 151 bp paired-end reads. Upon completion of the sequencing run, the quality of the raw data was assessed using Illumina’s software SAV (Sequence Analysis Viewer).

### Sequence Alignment and Variant Calling

Sequence alignment and variant calling were performed by HiSeq Analysis Software 2.0 (HAS2.0) (Illumina), according to the manufacturer’s protocol. The raw basecall (bcl) file of each sample was converted to FASTQ format with adapter trimming. The FASTQ reads were aligned to the reference human genome hg19 using Isaac software^20^ following the default setting. The variant calling was performed with BCFtools^21^ using confidently mapped reads only (mapping quality ≥30 and base quality ≥30). In the subsequent analyses, we used reliable sites (14,494,268 bp) of the total Y chromosomal length with a Phred quality score greater than 200 for more than 95% of the individuals.

### Phylogenetic Trees

A NJ^9^ tree of the 345 Japanese Y chromosomes was constructed using MEGA7^22^ based on the genetic distance matrix (p-distance) calculated from the 28,254 SNPs. The reliabilities of the NJ tree were confirmed using the bootstrap method with 1,000 replicates. The NJ tree of East Asian Y chromosomes was constructed using a total of 3,006 biallelic SNPs, that were polymorphic in each of the three datasets (i.e. 345 Japanese subjects in this study, 47 Koreans from The Personal Genome Project Korea (KPGP)^10^, and 244 East Asians (46 CHB, 52 CHS, 56 JPT, 46 KHV, and 44 CDX samples) from The 1000 Genomes Project Phase 3^11^). From the VCF files of the mainland Japanese population and the Korean population, a multi-fasta file of mtDNA sequences of 345 Japanese and 47 Koreans was created by vcf-consensus command of vcftools^23^, with hg19 as a reference. A NJ tree for mtDNA sequences was constructed based on the multi-fasta file by using MEGA7^22^ under the T92 model^24^.

### Genotyping

A SNP on the Y chromosome, rs2032626 (rs2032626-G allele is a marker for haplogroup D1b containing the YAP+ variant), was genotyped by using TaqMan assays (Applied Biosystems, Foster City, CA, USA) for 345 male and 80 female (as negative controls) Japanese subjects.

### Estimation of the Frequencies of the Seven Y-Chromosome Clades in the Jomon People

A phylogenetic analysis indicated the presence of seven Y-chromosome clades in the mainland Japanese population. It is not feasible to analytically estimate the frequencies of seven clades in the Jomon people. Instead, Monte Carlo simulation was performed in order to obtain the most probable frequencies. In the simulation, the clade frequencies in the mainland Japanese and the Koreans were assumed to be equal to those in the admixed population and in the Yayoi immigrants, respectively, at the time of the admixture (i.e. at the beginning of the Yayoi period). In each simulation run, a single uniformly distributed random number in the interval (0, 1), *r*_*i*_, was given for clade *i*. Then, the frequency of clade *i* in the Jomon people, *x*_*i*_, was calculated as:

$${x}_{i}=\frac{{r}_{i}}{{\sum }_{i=1}^{7}\,{r}_{i}}.$$

The observed frequencies of clade *i* in the mainland Japanese and the Koreans were denoted by *y*_*i*_ and *z*_*i*_. Assuming the admixture rate of 0.12 as suggested by a previous study^7^, the frequency of clade *i* in the admixed population, *w*_*i*_, was given by 0.12*x*_*i*_ + 0.88*z*_*i*_. The similarity between the simulated (admixed) and the actual (mainland Japanese) populations was evaluated by the similarity index (*SI*):

$$SI=\sum _{i=1}^{7}\frac{|{w}_{i}-{y}_{i}|}{{w}_{i}+{y}_{i}}$$

A set of *x*_*i*_ (*i* = 1 ~ 7) showing smaller *SI* is considered to be more likely. The above procedure was repeated 10^8^ times. From the 10^8^ sets of the clade frequencies in the Jomon people, 20 sets were selected in ascending order of *SI*, and then the average frequency of each clade was calculated for the 20 sets.

### Bayesian Coalescent Inference of Historical Change in Population Size

BSP analysis^8^, a Bayesian coalescent approach to inferring historical change from effective population size, using multiple haploid genomes undergoing uniparental inheritance without recombination, was applied separately to Y chromosomes of mainland Japanese in clades 1 and 3. Here, 7,834 and 7,252 SNPs observed in the mainland Japanese were used for clades 1 and 3, respectively. The input xml file was created by BEAUTi 2 program^25^. Then, BEAST 2.4.4 program^25^ was used for Bayesian inference of the change in population size using Markov chain Monte Carlo (MCMC) method. The chain length was set to a value such that the Effective Sample Size (ESS) reached 200. The mutation rate was set to 0.74 × 10^−9^ substitutions per site per year^18^. For the “clock rate” of the BEAUTi 2 input, the mutation rate was multiplied by “reliable sites” (14,494,268 bp) and divided by observed SNP numbers (7,834 bp for clade 1 and 7,252 bp for clade 3). The Jukes-Cantor (JC69) model was selected as a base substitution model. The log file was inspected in Tracer v1.5 (available from http://tree.bio.ed.ac.uk/software/tracer/) for convergence of the chain and ESS values, and the Bayesian Skyline Reconstruction was run.

### Coalescent Simulation Using fastsimcoal2

The fastsimcoal2^14^ simulation, that reproduces the relative changes in population size of the Jomon people obtained by the BSP of clade 1, was performed ten times. In the simulation, the population size was assumed to be increased 14,500 YBP, significantly decreased 3,000 YBP, and significantly increased 2,000 YBP. The number of SNPs on each gene genealogy was set to 28,254. In each simulation run, 200 chromosomes were sampled. The setting parameters were as follows:

//Number of population samples (demes)

1

//Population effective sizes (number of genes)

10000

//Sample sizes

200

//Growth rates: negative growth implies population expansion

0

//Number of migration matrices: 0 implies no migration between demes

0

//historical event: time, source, sink, migrants, new size, new growth rate, migr. matrix

3 historical event

80 0 0 0 0.112 0 0

120 0 0 0 3.58 0 0

580 0 0 0 0.172 0 0

//Number of independent loci [chromosome]

1 0

//Per chromosome: Number of linkage blocks

1

//per Block: data type, num loci, rec. rate and mut rate + optional parameters

SNP 28254 0 0

The output gene genealogy in each run was classified into two clades, and the BSP was performed only for the major clade (frequency more than 50%).

## Supplementary information

Supplementary figures

## Data Availability

The Japanese Y-chromosome data used in the BSP are available from the corresponding author upon request.
