# Supplementary material for: Analysis of whole Y-chromosome sequences reveals the Japanese population history in the Jomon period
Source: Sci Rep. 2019 Jun 17;9:8556. doi: 10.1038/s41598-019-44473-z (PMC6572846; doi:10.1038/s41598-019-44473-z)
Supplement: Supplementary file 1 — Supplementary figures [file 41598_2019_44473_MOESM1_ESM.pdf]

# Analysis of whole Y-chromosome sequences reveals the Japanese population history in the Jomon period

Yusuke Watanabe,[1] Izumi Naka,[1] Seik-Soon Khor,[2] Hiromi Sawai,[2] Yuki Hitomi,[2] Katsushi Tokunaga,[2] Jun Ohashi[1,*]

[1]Department of Biological Sciences, Graduate School of Science, The University of Tokyo, Tokyo 113-0033, Japan.
[2]Department of Human Genetics, Graduate School of Medicine, The University of Tokyo, Tokyo 113-0033, Japan.

*Correspondence: juno-tky@umin.ac.jp (J.O.)

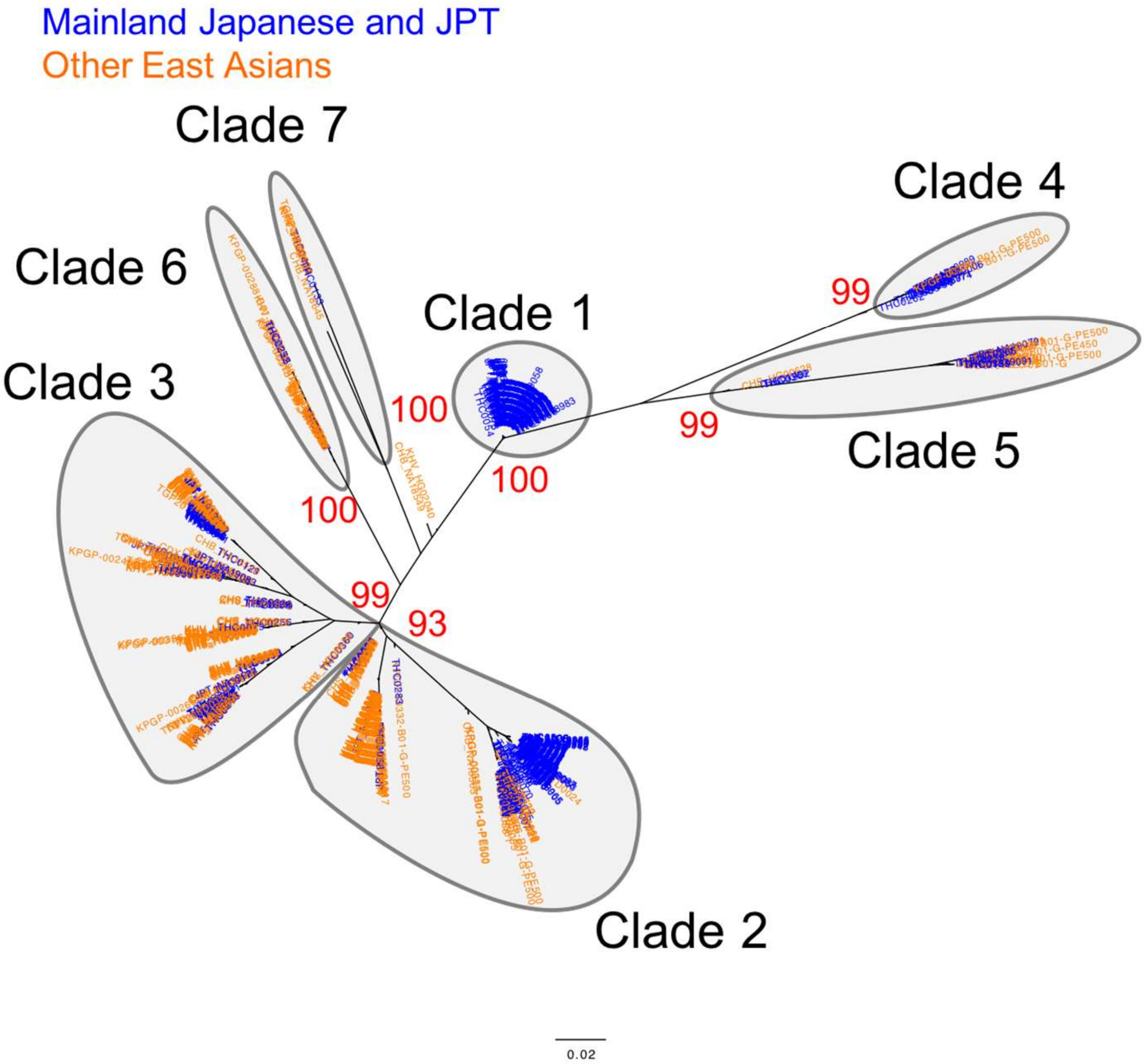


**Figure S1. Neighbor-joining tree of East Asian Y chromosomes**
The neighbor-joining (NJ) tree of 345 mainland Japanese, 45 Korean and 244 East Asian Y chromosomes was constructed based on 3,006 Y- chromosomal single nucleotide polymorphisms (SNPs) using MEGA7. Since mainland Japanese-specific SNPs are not included in this analysis, the branch lengths of the NJ tree are not correct. Bootstrap values (%) for seven clades are shown in red. Y-chromosome data of five East Asian populations were obtained from the 1000 Genomes Project phase 3. JPT: Japanese in Tokyo, Japan; CHB: Han Chinese in Beijing, China; CHS: Southern Han Chinese; CDX: Chinese Dai in Xishuangbanna, China; and KHV: Kinh in Ho Chi Minh City, Vietnam.

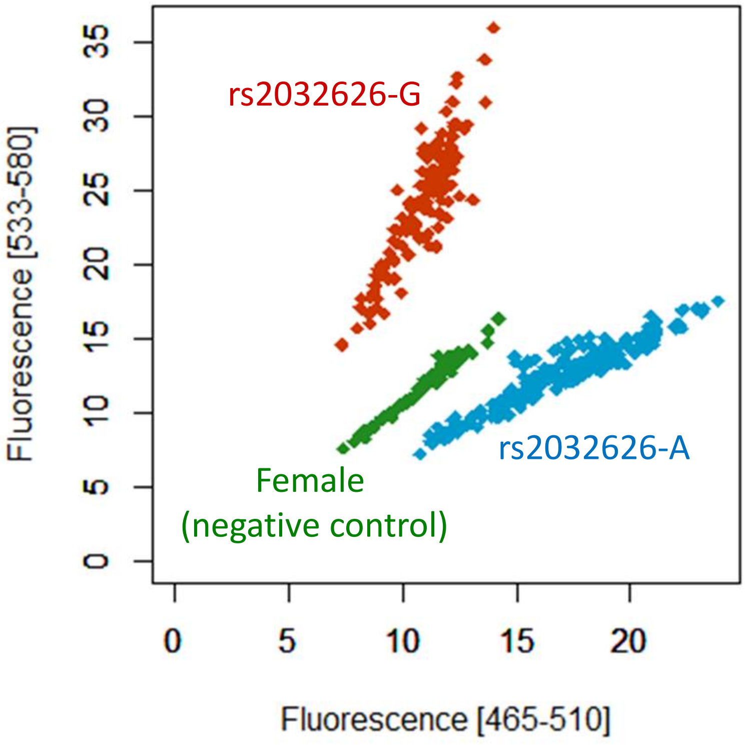


**Figure S2. TaqMan genotyping assay for rs2032626**
The genotypes of rs2032626-G and rs2032626-A are indicated by red and blue diamonds. The female samples (genotyped as negative controls) are indicated by green diamonds.

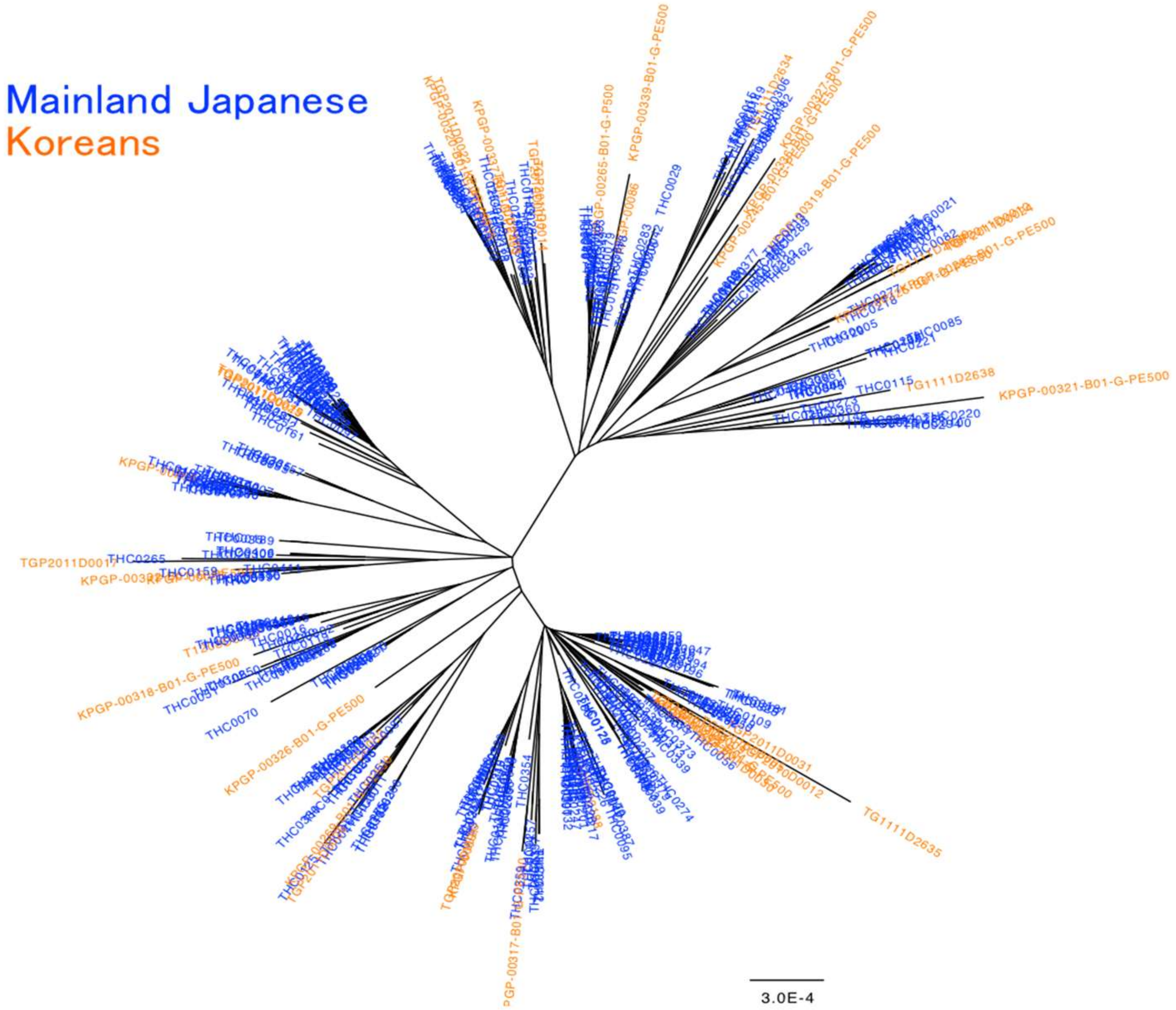


**Figure S3. Neighbor-joining tree of whole mitochondrial DNA sequences from 345 Mainland Japanese and 45 Koreans**

The neighbor-joining tree was reconstructed using MEGA7.

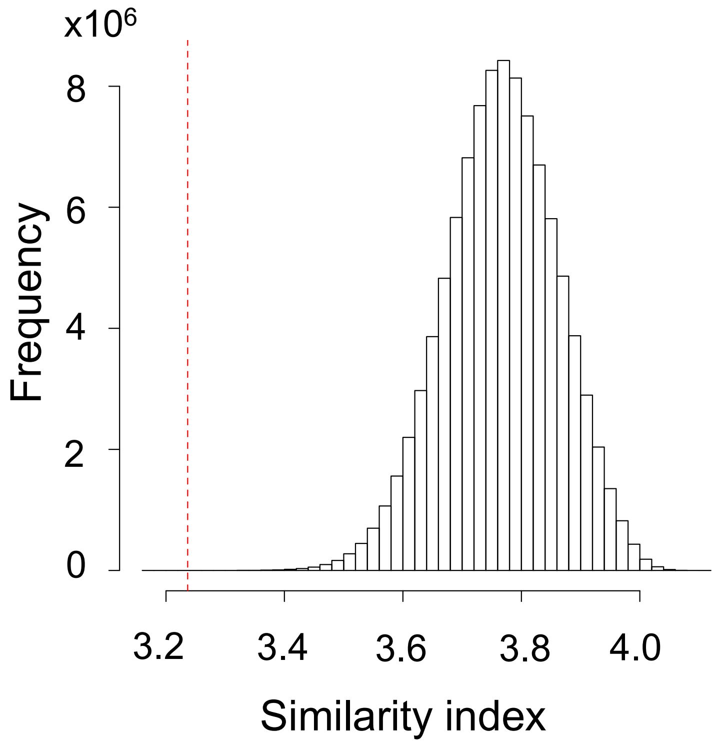


**Figure S4. The distribution of similarity index (SI) for $10^8$ simulated data sets of the clade frequencies in Jomon population**

The red dashed line indicates the 20th smallest SI value (SI=3.24).

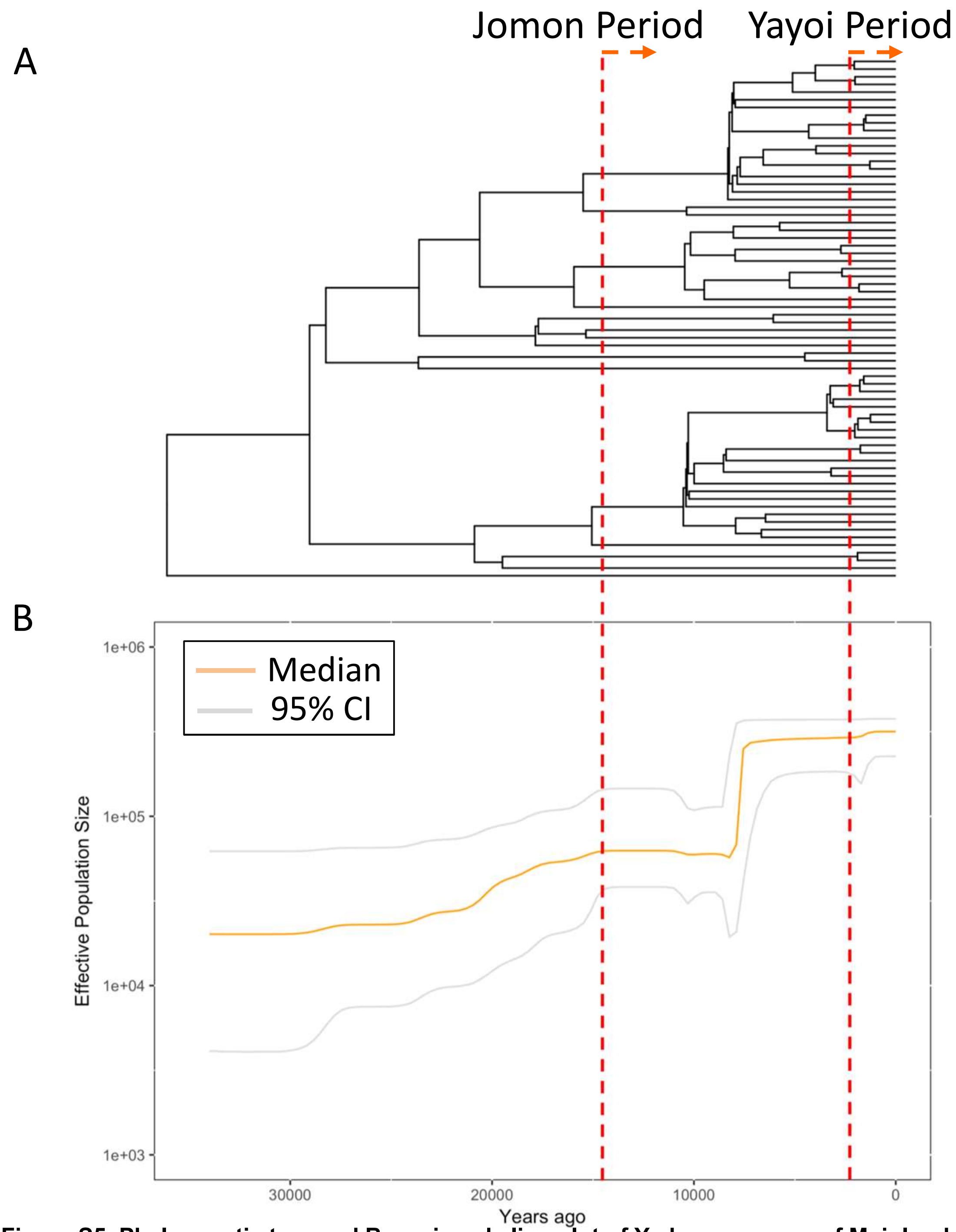


**Figure S5. Phylogenetic tree and Bayesian skyline plot of Y chromosomes of Mainland Japanese in clade 3**

(A) Phylogenetic tree of clade 3 estimated by BEAST 2.4.4. (B) BSP of 67 Y chromosomes of mainland Japanese in clade 3. BEAST 2.4.4 was used to obtain the plot. The red dashed lines represent 14,500 years ago (the beginning of Jomon period) and 2,300 years ago (the beginning of Yayoi period).
